# Supplementary material for: CXCR4 involvement in neurodegenerative diseases
Source: Transl Psychiatry. 2018 Apr 11;8:73. doi: 10.1038/s41398-017-0049-7 (PMC5893558; doi:10.1038/s41398-017-0049-7)
Supplement: Supplementary file 1 — Supplemental Materials [file 41398_2017_49_MOESM1_ESM.docx]

**SUPPLEMENTAL METHODS**

**Genomic Control**

The empirical null distribution in GWAS has been shown to be altered by global variance inflation due to underlying population stratification and cryptic relatedness^1^ and deflation due to over-correction of test statistics for polygenic traits by standard genomic control techniques^2^. We utilized a control method that leverages only intergenic SNPs, which are likely depleted for true associations^3^. First, we annotated the SNPs to genic (5’UTR, exon, intron, 3’UTR) and intergenic regions using information from the 1,000 genomes project (1KGP). For each phenotype, we estimated the genomic inflation factor λGC given the effect sizes of associations with intergenic SNPs. The inflation factor, λGC is the median of non-central parameters of chi-square statistics divided by the expected median of a chi-square distribution with one degree of freedom. Once inflation factor is derived, we then divided all test statistics by λGC.

**Conjunction statistics – test of association with both phenotypes**

We defined the conjunction statistics (denoted as FDR Trait1 & Trait2) as the maximum of the conditional FDR in both directions, i.e. FDR Trait1 & Trait2 = max(FDR Trait1 | Trait2, FDR Trait2 | Trait1) based on the combination of p-value for the SNP in PSP and PD, FTD, or AD ^4,5^. The FDR given trait pairs is estimated through interpolating the observed conditional effects into 2D look-up table. The conjunction statistic allows for identification of SNPs that are associated with both phenotypes, which minimizes the effect of a single phenotype driving the common association signal. Table 2 lists all SNPs with conjunction FDR < 0.05 (-log10(FDR) > 1.3) with PSP and PD, FTD, and AD after removing all SNPs with r2 > 0.2 based on 1KGP linkage disequilibrium (LD) (pruning).

**Conjunction FDR Manhattan plots**

To illustrate the localization of the genetic markers associated with PSP given PD, FTD, or AD we used a ‘Conjunction FDR Manhattan plot’, plotting all SNPs within an LD block in relation to their chromosomal location. As illustrated in Figure 1b within the main manuscript, the large points represent the SNPs with FDR < 0.05, whereas the small points represent the non-significant SNPs. All SNPs before ‘pruning’ (removing all SNPs with r2 > 0.2 based on 1KGP LD structure) are shown. The strongest signal in each LD block is illustrated with a black line around the circles. This was identified by ranking all SNPs in increasing order, based on the conditional FDR value for PSP, and then removing SNPs in LD r2 > 0.2 with any higher ranked SNP. Thus, the selected locus was the most significantly associated with PSP in each LD block for PD, FTD, or AD.

**IGAP Cohort**

International Genomics of Alzheimer's Project (IGAP) is a large two-stage study based upon genome-wide association studies (GWAS) on individuals of European ancestry. In stage 1, IGAP used genotyped and imputed data on 7,055,881 single nucleotide polymorphisms (SNPs) to meta-analyse four previously-published GWAS datasets consisting of 17,008 Alzheimer's disease cases and 37,154 controls (The European Alzheimer's disease Initiative – EADI the Alzheimer Disease Genetics Consortium – ADGC The Cohorts for Heart and Aging Research in Genomic Epidemiology consortium – CHARGE The Genetic and Environmental Risk in AD consortium – GERAD). In stage 2, 11,632 SNPs were genotyped and tested for association in an independent set of 8,572 Alzheimer's disease cases and 11,312 controls. Finally, a meta-analysis was performed combining results from stages 1 & 2. In this study, we focused on the stage 1 IGAP SNPs.

**SUPPLEMENTAL RESULTS**

*Relationship between CXCR4, TMEM119 and AIF1 in transgenic mouse model*

Within the hippocampus, across all evaluated time points (2,4,6 and 18 months) for wild-type and Tau-P301L mice, we found a relationship between *CXCR4* and *TMEM119* (Pearson’s R = 0.52, p = 5.3 x 10^-5^) and *AIF1* (Pearson’s R = 0.52, p = 5.2 x 10^-5^). Within the cortex, we found no relationship between *CXCR4* and *TMEM119* (Pearson’s R = 0.17, p = 0.21) and *AIF1* (Pearson’s R = 0.13, p = 0.32). Within the cerebellum, we found no relationship between *CXCR4* and *TMEM119* (Pearson’s R = 0.20, p = 0.13) and *AIF1* (0.05, p = 0.68).

*Relationship between CXCR4 associated genes, TMEM119 and AIF1 in transgenic mouse model*

We also assessed the relationship between *CXCR4* associated genes (*CXCL12*, *RALB, CCR5 and TLR2*) and *TMEM119* and *AIF1*. Within the hippocampus, across all evaluated time points (2,4,6 and 18 months) for wild-type and Tau-P301L mice, we found a relationship between *CCR5* and *TMEM119* (Pearson’s R = 0.58, p = 3.5 x 10^-6^) and *AIF1* (Pearson’s R = 0.63, p = 2.4 x 10^-7^). Similarly within the hippocampus, we also found a relationship between *TLR2* and *TMEM119* (Pearson’s R = 0.71, p = 1.3 x 10^-9^) and *AIF1* (Pearson’s R = 0.86, p = 2.2 x 10^-16^). In contrast we found no association between *TMEM119* and *AIF1* and *CXCL12* (Pearson’s R = -0.119, p = 0.38 and Pearson’s R = -0.05, p = 0.69, respectively), and *RALB* (Pearson’s R = 0.13, p = 0.35 and Pearson’s R = 0.23, p = 0.09, respectively) within the hippocampus.

Within the cortex, we found a significant association between *TMEM119* and *AIF1* and *TLR2* (Pearson’s R = 0.51, p = 6.8 x 10^-5^ and Pearson’s R = 0.83, p = 1.34 x 10^-15^, respectively), and *CCR5* (Pearson’s R = 0.58, p = 2.3 x 10^-6^ and Pearson’s R = 0.55, p = 1.4 x 10^-5^, respectively). In contrast we found no relationship between *TMEM119* and *AIF1* and either *RALB* or *CXCL12* (all p-values > 0.05) within the cortex.

Within the cerebellum, we found a significant association between *TMEM119* and *AIF1* and *TLR2* (Pearson’s R = 0.64, p = 1.1 x 10^-7^ and Pearson’s R = 0.86, p = < 2.2 x 10^-16^, respectively), and *CCR5* (Pearson’s R = 0.63, p = 2.3 x 10^-7^ and Pearson’s R = 0.68, p = 9.4 x 10^-9^, respectively). In contrast we found no relationship between *TMEM119* and *AIF1* and either *RALB* or *CXCL12* (all p-values > 0.05) within the cerebellum.

*TMEM119 and AIF1 expression is elevated in tau transgenic mouse model*

Within the hippocampus, *TMEM119* (F = 42.9, p = 3.9 x 10^-8^) and *AIF1* (F = 149.6, p = < 2.0 x 10^-16^) were elevated over time. Within the cortex, we found that *AIF1* (F = 12.7, p = 0.008) was elevated over time; in contrast, *TMEM119* expression was not altered over time (F = 1.1, p = 0.29). Within the cerebellum, we found that expression of *TMEM119* (F = 2.7, p = 0.11) and *AIF1* (F = 2.1, p = 0.15) was not altered over time.

**SUPPLEMENTAL REFERENCES**

1 Devlin B, Roeder K. Genomic control for association studies. *Biometrics* 1999; **55**: 997–1004.

2 Yang J, Weedon MN, Purcell S, Lettre G, Estrada K, Willer CJ *et al.* Genomic inflation factors under polygenic inheritance. *Eur J Hum Genet* 2011; **19**: 807–812.

3 Schork AJ, Thompson WK, Pham P, Torkamani A, Roddey JC, Sullivan PF *et al.* All SNPs are not created equal: genome-wide association studies reveal a consistent pattern of enrichment among functionally annotated SNPs. *PLoS Genet* 2013; **9**: e1003449.

4 Efron B. *Large-scale inference : empirical Bayes methods for estimation, testing, and prediction*. Cambridge University Press: New York, 2010.

5 Schweder T, Spjøtvoll E. Plots of p-values to evaluate many tests simultaneously. *Biometrika* 1982; **69**: 493–502.

**SUPPLEMENTAL FIGURES**

**Supplemental Figure 1.** Linkage disequilbrium within PSP and PD risk loci. **(a)** Plot for rs749873. **(b)** Plot for rs199533.

**Supplemental Figure 2.** Line plots illustrating *MAPT* gene expression in tau transgenic (red line) and wild-type mice (black line) from 2 to 18 months of age, across the (a) hippocampus, (b) cortex, and (c) cerebellum. Total tau pathology (d) over time is also illustrated.

**Supplemental Figure 3.** Line plots illustrating *TLR2* gene expression in tau transgenic (red line) and wild-type mice (black line) from 2 to 18 months of age, across the (a) hippocampus, (b) cortex, and (c) cerebellum. Total tau pathology (d) over time is also illustrated.

**Supplemental Figure 4.** Line plots illustrating *CCR5* gene expression in tau transgenic (red line) and wild-type mice (black line) from 2 to 18 months of age, across the (a) hippocampus, (b) cortex, and (c) cerebellum. Total tau pathology (d) over time is also illustrated.

**Supplemental Figure 5.** Line plots illustrating *CXCL12* gene expression in tau transgenic (red line) and wild-type mice (black line) from 2 to 18 months of age, across the (a) hippocampus, (b) cortex, and (c) cerebellum. Total tau pathology (d) over time is also illustrated.

**Supplemental Figure 6.** Line plots illustrating *RALB* gene expression in tau transgenic (red line) and wild-type mice (black line) from 2 to 18 months of age, across the (a) hippocampus, (b) cortex, and (c) cerebellum. Total tau pathology (d) over time is also illustrated.

**Supplemental Figure 1a**

**Supplemental Figure 1b**

**Supplemental Figure 2**

**
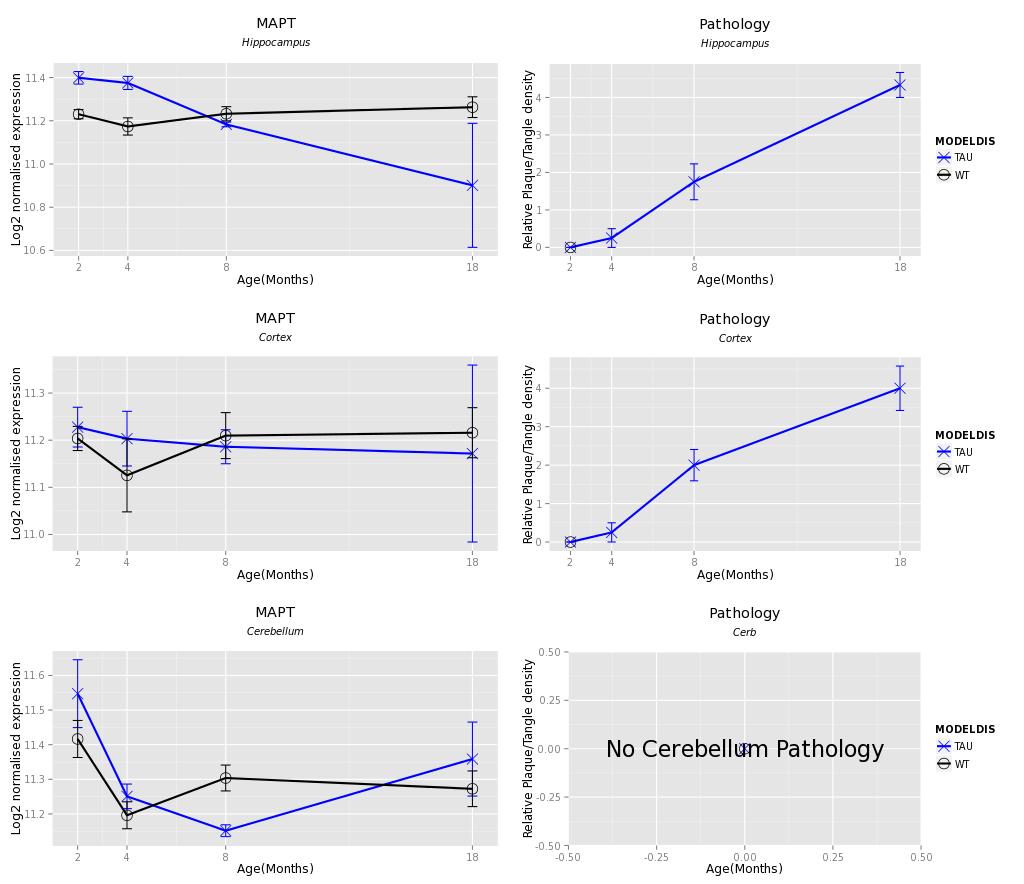
**

**Supplemental Figure 3**

**
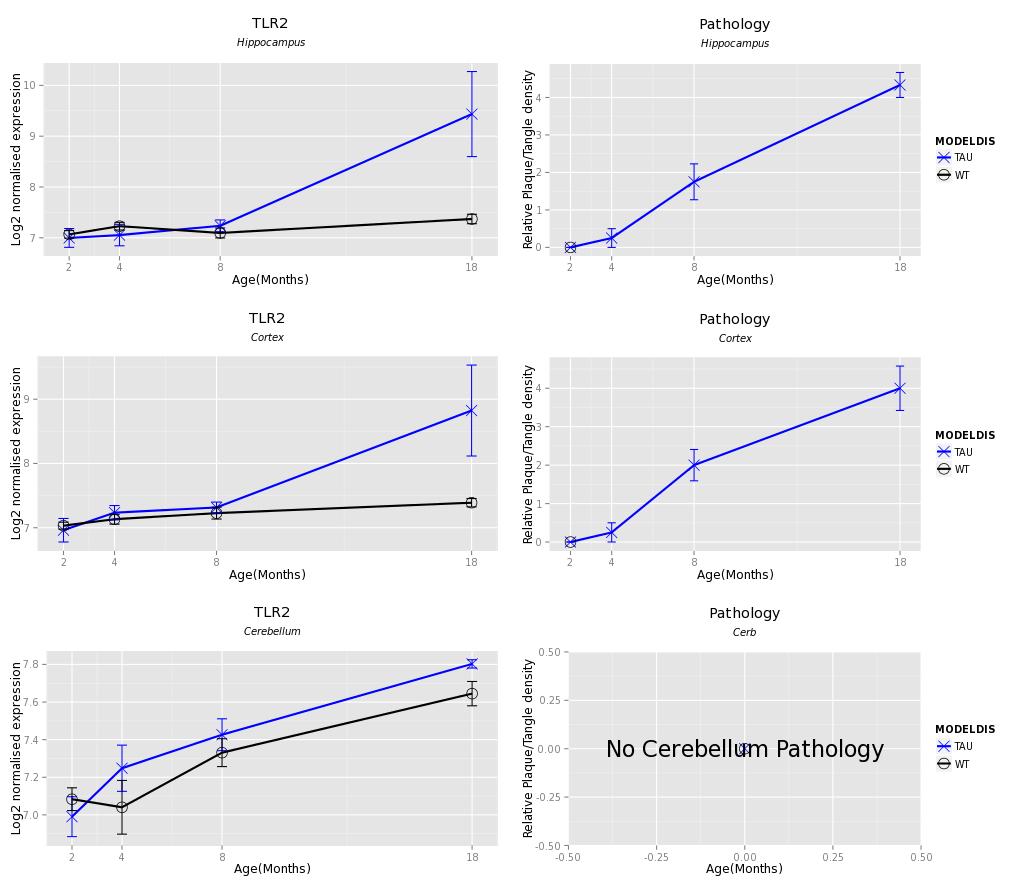
**

**Supplemental Figure 4**

**
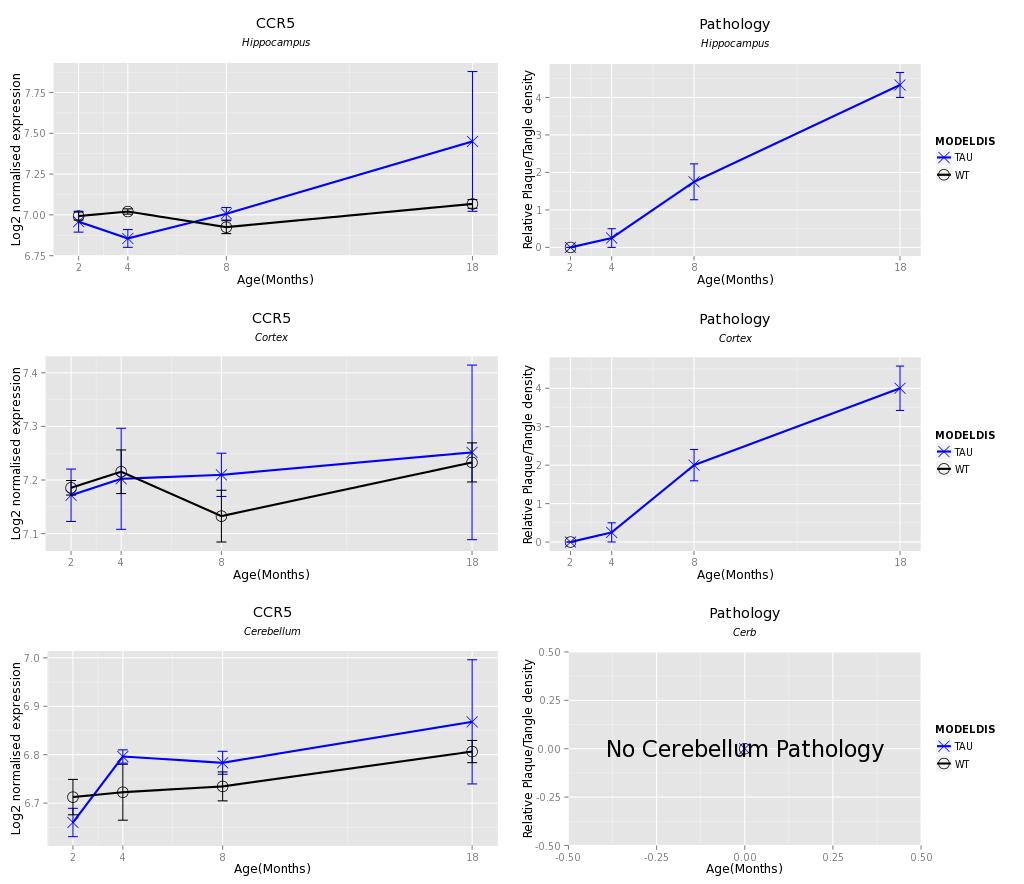
**

**Supplemental Figure 5**

**
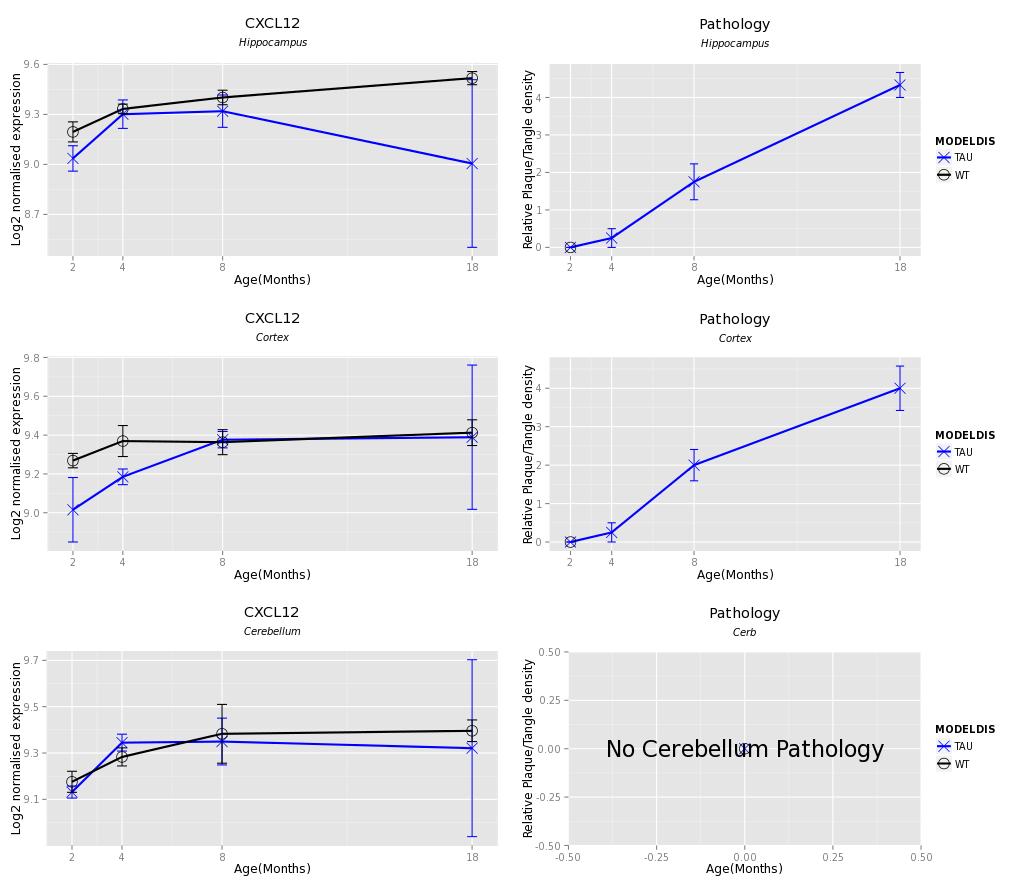
**

**Supplemental Figure 6**


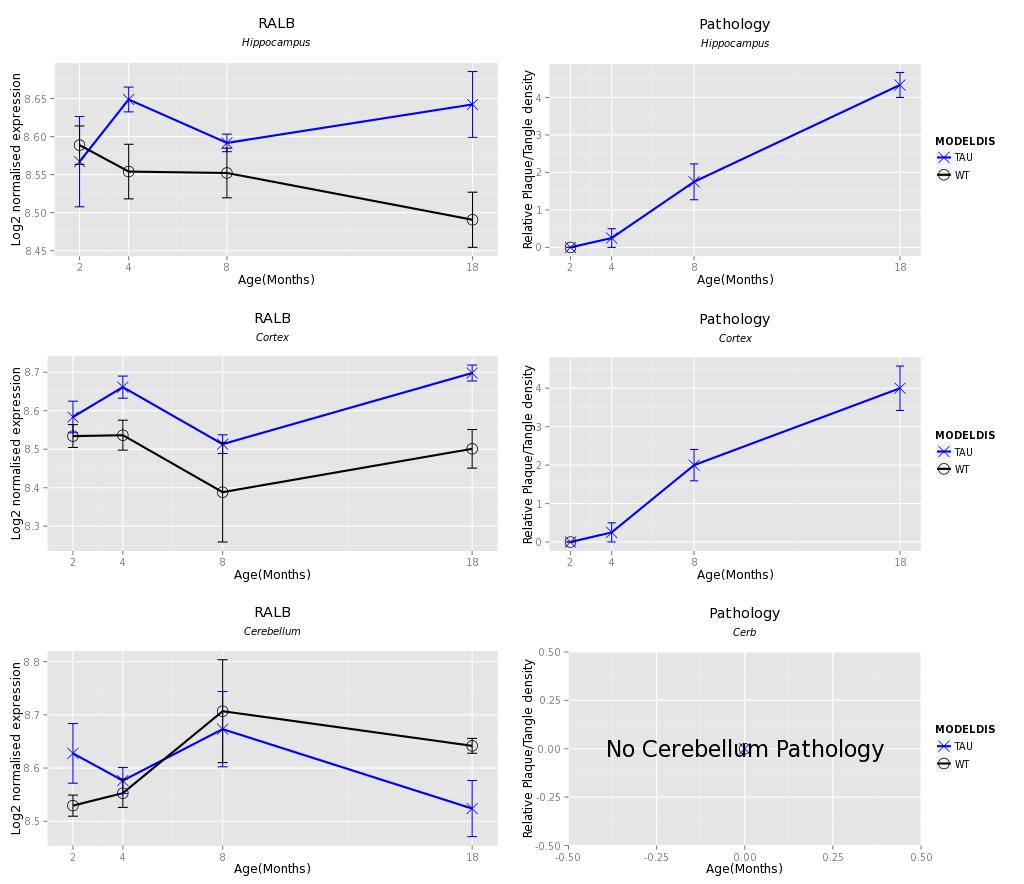


**SUPPLEMENTAL ACKNOWLEDGEMENTS**

**IGAP:** We thank the International Genomics of Alzheimer's Project (IGAP) for providing summary results data for these analyses. The investigators within IGAP contributed to the design and implementation of IGAP and/or provided data but did not participate in analysis or writing of this report. IGAP was made possible by the generous participation of the control subjects, the patients, and their families. The i–Select chips was funded by the French National Foundation on Alzheimer's disease and related disorders. EADI was supported by the LABEX (laboratory of excellence program investment for the future) DISTALZ grant, Inserm, Institut Pasteur de Lille, Université de Lille 2 and the Lille University Hospital. GERAD was supported by the Medical Research Council (Grant n° 503480), Alzheimer's Research UK (Grant n° 503176), the Wellcome Trust (Grant n° 082604/2/07/Z) and German Federal Ministry of Education and Research (BMBF): Competence Network Dementia (CND) grant n° 01GI0102, 01GI0711, 01GI0420. CHARGE was partly supported by the NIH/NIA grant R01 AG033193 and the NIA AG081220 and AGES contract N01–AG–12100, the NHLBI grant R01 HL105756, the Icelandic Heart Association, and the Erasmus Medical Center and Erasmus University. ADGC was supported by the NIH/NIA grants: U01 AG032984, U24 AG021886, U01 AG016976, and the Alzheimer's Association grant ADGC–10–196728.

**ADGC:** The National Institutes of Health, National Institute on Aging (NIH-NIA) supported this work through the following grants: ADGC, U01 AG032984, RC2 AG036528; NACC, U01 AG016976; NCRAD, U24 AG021886; NIA LOAD, U24 AG026395, U24 AG026390; Banner Sun Health Research Institute P30 AG019610; Boston University, P30 AG013846, U01 AG10483, R01 CA129769, R01 MH080295, R01 AG017173, R01 AG025259, R01AG33193; Columbia University, P50 AG008702, R37 AG015473; Duke University, P30 AG028377, AG05128; Emory University, AG025688; Group Health Research Institute, UO1 AG06781, UO1 HG004610; Indiana University, P30 AG10133; Johns Hopkins University, P50 AG005146, R01 AG020688; Massachusetts General Hospital, P50 AG005134; Mayo Clinic, P50 AG016574; Mount Sinai School of Medicine, P50 AG005138, P01 AG002219; New York University, P30 AG08051, MO1RR00096, UL1 RR029893, 5R01AG012101, 5R01AG022374, 5R01AG013616, 1RC2AG036502, 1R01AG035137; Northwestern University, P30 AG013854; Oregon Health & Science University, P30 AG008017, R01 AG026916; Rush University, P30 AG010161, R01 AG019085, R01 AG15819, R01 AG17917, R01 AG30146; TGen, R01 NS059873; University of Alabama at Birmingham, P50 AG016582, UL1RR02777; University of Arizona, R01 AG031581; University of California, Davis, P30 AG010129; University of California, Irvine, P50 AG016573, P50, P50 AG016575, P50 AG016576, P50 AG016577; University of California, Los Angeles, P50 AG016570; University of California, San Diego, P50 AG005131; University of California, San Francisco, P50 AG023501, P01 AG019724; University of Kentucky, P30 AG028383, AG05144; University of Michigan, P50 AG008671; University of Pennsylvania, P30 AG010124; University of Pittsburgh, P50 AG005133, AG030653; University of Southern California, P50 AG005142; University of Texas Southwestern, P30 AG012300; University of Miami, R01 AG027944, AG010491, AG027944, AG021547, AG019757; University of Washington, P50 AG005136; Vanderbilt University, R01 AG019085; and Washington University, P50 AG005681, P01 AG03991. The Kathleen Price Bryan Brain Bank at Duke University Medical Center is funded by NINDS grant # NS39764, NIMH MH60451 and by Glaxo Smith Kline. Genotyping of the TGEN2 cohort was supported by Kronos Science. The TGen series was also funded by NIA grant AG034504 to AJM, The Banner Alzheimer’s Foundation, The Johnnie B. Byrd Sr. Alzheimer’s Institute, the Medical Research Council, and the state of Arizona and also includes samples from the following sites: Newcastle Brain Tissue Resource (funding via the Medical Research Council, local NHS trusts and Newcastle University), MRC London Brain Bank for Neurodegenerative Diseases (funding via the Medical Research Council),South West Dementia Brain Bank (funding via numerous sources including the Higher Education Funding Council for England (HEFCE), Alzheimer’s Research Trust (ART), BRACE as well as North Bristol NHS Trust Research and Innovation Department and DeNDRoN), The Netherlands Brain Bank (funding via numerous sources including Stichting MS Research, Brain Net Europe, Hersenstichting Nederland Breinbrekend Werk, International Parkinson Fonds, Internationale Stiching Alzheimer Onderzoek), Institut de Neuropatologia, Servei Anatomia Patologica, Universitat de Barcelona. ADNI Funding for ADNI is through the Northern California Institute for Research and Education by grants from Abbott, AstraZeneca AB, Bayer Schering Pharma AG, Bristol-Myers Squibb, Eisai Global Clinical Development, Elan Corporation, Genentech, GE Healthcare, GlaxoSmithKline, Innogenetics, Johnson and Johnson, Eli Lilly and Co., Medpace, Inc., Merck and Co., Inc., Novartis AG, Pfizer Inc, F. Hoffman-La Roche, Schering-Plough, Synarc, Inc., Alzheimer's Association, Alzheimer's Drug Discovery Foundation, the Dana Foundation, and by the National Institute of Biomedical Imaging and Bioengineering and NIA grants U01 AG024904, RC2 AG036535, K01 AG030514. We thank Drs. D. Stephen Snyder and Marilyn Miller from NIA who are *ex-officio* ADGC members. Support was also from the Alzheimer’s Association (LAF, IIRG-08-89720; MP-V, IIRG-05-14147) and the US Department of Veterans Affairs Administration, Office of Research and Development, Biomedical Laboratory Research Program. P.S.G.-H. is supported by Wellcome Trust, Howard Hughes Medical Institute, and the Canadian Institute of Health Research. Data for this study were prepared, archived, and distributed by the National Institute on Aging Alzheimer’s Disease Data Storage Site (NIAGADS) at the University of Pennsylvania (U24-AG041689-01), funded by the National Institute on Aging.

**The International FTD-Genomics Consortium (IFGC)**

**Acknowledgments**

Intramural funding from the National Institute of Neurological Disorders and Stroke (NINDS) and National Institute on Aging (NIA), the Wellcome/MRC Centre on Parkinson’s disease, Alzheimer’s Research UK (ARUK, Grant ARUK-PG2012-18) and by the office of the Dean of the School of Medicine, Department of Internal Medicine, at Texas Tech University Health Sciences Center.

We thank Mike Hubank and Kerra Pearce at the Genomic core facility at the Institute of Child Health (ICH), University College of London (UCL), for assisting RF in performing Illumina genotyping experiments (FTD-GWAS genotyping). This study utilized the high-performance computational capabilities of the Biowulf Linux cluster at the National Institutes of Health, Bethesda, Md. (<http://biowulf.nih.gov>). North American Brain Expression Consortium (NABEC) - The work performed by the North American Brain Expression Consortium (NABEC) was supported in part by the Intramural Research Program of the National Institute on Aging, National Institutes of Health, part of the US Department of Health and Human Services; project number ZIA AG000932-04. In addition this work was supported by a Research Grant from the Department of Defense, W81XWH-09-2-0128. UK Brain Expression Consortium (UKBEC) - This work performed by the UK Brain Expression Consortium (UKBEC) was supported by the MRC through the MRC Sudden Death Brain Bank (C.S.), by a Project Grant (G0901254 to J.H. and M.W.) and by a Fellowship award (G0802462 to M.R.). D.T. was supported by the King Faisal Specialist Hospital and Research Centre, Saudi Arabia. Computing facilities used at King's College London were supported by the National Institute for Health Research (NIHR) Biomedical Research Centre based at Guy's and St Thomas' NHS Foundation Trust and King's College London. We would like to thank AROS Applied Biotechnology AS company laboratories and Affymetrix for their valuable input. RF’s work is supported by Alzheimer’s Society, UK; JBJK was supported by the National Health and Medical Resarch Council (NHMRC) Australia, Project Grants 510217 and 1005769; CDS was supported by NHMRC Project Grants 630428 and 1005769; PRS was supported by NHMRC Project Grants 510217 and 1005769 and acknowledges that DNA samples were prepared by Genetic Repositories Australia, supported by NHMRC Enabling Grant 401184; GMH was supported by NHMRC Research Fellowship 630434, Project Grant 1029538, Program Grant 1037746; JRH was supported by the Australian Research Council Federation Fellowship, NHMRC Project Grant 1029538, NHMRC Program Grant 1037746; OP was supported by NHMRC Career Development Fellowship 1022684, Project Grant 1003139. IH, AR and MB acknowledge the patients and controls who participated in this project and the Trinitat Port-Carbó and her family who are supporting Fundació ACE research programs. CC was supported by Grant P30-NS069329-01 and acknowledges that the recruitment and clinical characterization of research participants at Washington University were supported by NIH P50 AG05681, P01 AG03991, and P01 AG026276. LB and GB were supported by the Ricerca Corrente, Italian Ministry of Health; RG was supported by Fondazione CARIPLO 2009-2633, Ricerca Corrente, Italian Ministry of Health; GF was supported by Fondazione CARIPLO 2009-2633. ES was supported by the Italian Ministry of Health; CF was supported by Fondazione Cariplo; MS was supported from the Italian Ministry of Health (Ricerca Corrente); MLW was supported by Government funding of clinical research within NHS Sweden (ALF); KN was supported by Thure Carlsson Foundation; CN was supported by Swedish Alzheimer Fund. IRAM and GYRH were supported by CIHR (grant 74580) PARF (grant C06-01). JG was supported by the NINDS intramural research funds for FTD research. CMM was supported by Medical Research Council UK, Brains for Dementia Research, Alzheimer's Society, Alzheimer's Research UK, National Institutes for Health Research, Department of Health, Yvonne Mairy Bequest and acknowledges that tissue made available for this study was provided by the Newcastle Brain Tissue Resource, which was funded in part by grants G0400074 and G1100540 from the UK MRC, the Alzheimer’s Research Trust and Alzheimer’s Society through the Brains for Dementia Research Initiative and an NIHR Biomedical Research Centre Grant in Ageing and Health, and NIHR Biomedical Research Unit in Lewy Body Disorders. CMM was supported by the UK Department of Health and Medical Research Council and the Research was supported by the National Institute for Health Research Newcastle Biomedical Research Centre based at Newcastle Hospitals Foundation Trust and Newcastle University and acknowledges that the views expressed are those of the authors and not necessarily those of the NHS, the NIHR or the Department of Health; JA was supported by MRC, Dunhill Medical Trust, Alzheimer's Research UK; TDG was supported by Wellcome Trust Senior Clinical Fellow; IGM was supported by NIHR Biomedical Research Centre and Unit on Ageing Grants and acknowledges the National Institute for Health Research Newcastle Biomedical Research Centre based at Newcastle Hospitals Foundation Trust and Newcastle University. The views expressed are those of the author(s) and not necessarily those of the NHS, the NIHR or the Department of Health; AJT was supported by Medical Research Council, Alzheimer's Society, Alzheimer's Research UK, National Institutes for Health Research. EJ was supported by NIHR, Newcastle Biomedical Research Centre. PP, CR, SOC and EA were supported partially by FIMA (Foundation for Applied Medical Research); PP acknowledges Manuel Seijo-Martínez (Department of Neurology, Hospital do Salnés, Pontevedra, Spain), Ramon Rene, Jordi Gascon and Jaume Campdelacreu (Department of Neurology, Hospital de Bellvitge, Barcelona, Spain) for providing FTD DNA samples. RP, JDS, PA and AK were supported by German Federal Ministry of Education and Research (BMBF; grant number FKZ 01GI1007A – German FTLD consortium). IR was supported by Ministero dell'Istruzione, dell'Università e della Ricerca (MIUR) of Italy. PStGH was supported by the Canadian Institutes of Health Research, Wellcome Trust, Ontario Research Fund. FT was supported by the Italian Ministry of Health (ricerca corrente) and MIUR grant RBAP11FRE9; GR and GG were supported by the Italian Ministry of Health (ricerca corrente). JBR was supported by Camrbidge NIHR Biomedical Research Centre and Wellcome Trust (088324). JU, JC, SM were supported by the MRC Prion Unit core funding and acknowledge MRC UK, UCLH Biomedical Research Centre, Queen Square Dementia BRU; SM acknowledges the work of John Beck, Tracy Campbell, Gary Adamson, Ron Druyeh, Jessica Lowe, Mark Poulter. AD acknowledges the work of Benedikt Bader and of Manuela Neumann, Sigrun Roeber, Thomas Arzberger and Hans Kretzschmar†; VMVD and JQT were supported by Grants AG032953, AG017586 and AG010124; MG was supported by Grants AG032953, AG017586, AG010124 and NS044266; VMVD acknowledges EunRan Suh, PhD for assistance with sample handling and Elisabeth McCarty-Wood for help in selection of cases; JQT acknowledges Terry Schuck, John Robinson and Kevin Raible for assistance with neuropathological evaluation of cases. CVB and the Antwerp site were in part funded by the MetLife Foundation for Medical Research Award (to CVB), the Belgian Science Policy Office (BELSPO) Interuniversity Attraction Poles program; the Alzheimer Research Foundation (SAO-FRA); the Medical Foundation Queen Elisabeth (GSKE); the Flemish Government initiated Methusalem Excellence Program (to CVB); the Research Foundation Flanders (FWO) and the University of Antwerp Research Fund.. CVB, MC and JvdZ acknowledge the neurologists S Engelborghs, PP De Deyn, A Sieben, R Vandenberghe and the neuropathologist JJ Martin for the clinical and pathological diagnoses. CVB, MC and JvdZ further thank the personnel of the Genetic Service Facility of the VIB Department of Molecular Genetics (<http://www.vibgeneticservicefacility.be>) and the Antwerp Biobank of the Institute Born-Bunge for their expert support. IL and AB were supported by the program “Investissements d’avenir” ANR-10-IAIHU-06 and acknowledges the contribution of The French research network on FTLD/FTLD-ALS for the contribution in samples collection. BN is founded by Fondazione Cassa di Risparmio di Pistoia e Pescia (grant 2014.0365), SS is founded by the Cassa di Risparmio di Firenze (grant 2014.0310) and a grant from Ministry of Health n° RF-2010-2319722. JEN was supported by the Novo Nordisk Foundation, Denmark. MR was supported by the German National Genome Network (NGFN); German Ministry for Education and Research Grant Number 01GS0465. JDR, MNR, NCF and JDW were supported by an MRC programme grant, the NIHR Queen Square Dementia Biomedical Research Unit (BRU) and the Leonard Wolfson Experimental Neurology Centre. MGS was supported by MRC grant n G0301152, Cambridge Biomedical Research Centre and acknowledges Mrs K Westmore for extracting DNA. HM was supported by the Motor Neuron Disease Association (Grant 6057). RR was supported by P50 AG016574, R01 NS080882, R01 NS065782, P50 NS72187 and the Consortium for Frontotemporal Dementia; DWD was supported by P50NS072187, P50AG016574, State of Florida Alzheimer Disease Initiative, & CurePSP, Inc.; NRGR, JEP, RCP, DK, BFB were supported by P50 AG016574; KAJ was supported by R01 AG037491; WWS was supported by NIH AG023501, AG019724, Consortium for Frontotemporal Dementia Research; BLM was supported by P50AG023501, P01AG019724, Consortium for FTD Research; HR was supported by AG032306. JCvS was supported by Stichting Dioraphte Foundation (11 02 03 00), Nuts Ohra Foundation (0801-69), Hersenstichting Nederland (BG 2010-02) and Alzheimer Nederland. CG and HHC acknowledge families, patients, clinicians including Dr Inger Nennesmo and Dr Vesna Jelic, Professor Laura Fratiglioni for control samples and Jenny Björkström, Håkan Thonberg, Charlotte Forsell, Anna-Karin Lindström and Lena Lilius for sample handling. CG was supported by Swedish Brain Power (SBP), the Strategic Research Programme in Neuroscience at Karolinska Institutet (StratNeuro), the regional agreement on medical training and clinical research (ALF) between Stockholm County Council and Karolinska Institutet, Swedish Alzheimer Foundation, Swedish Research Council, Karolinska Institutet PhD-student funding, King Gustaf V and Queen Victoria’s Free Mason Foundation. FP, AR, VD and FL acknowledge Labex DISTALZ. RF acknowledges the help and support of Mrs. June Howard at the Texas Tech University Health Sciences Center Office of Sponsored Programs for tremendous help in managing Material Transfer Agreement at TTUHSC.

**IFGC members and affiliations**

Raffaele Ferrari (UCL, Department of Molecular Neuroscience, Russell Square House, 9-12 Russell Square House, London, WC1B 5EH), Dena G Hernandez (Laboratory of Neurogenetics, National Institute on Aging, National Institutes of Health, Building 35, Room 1A215, 35 Convent Drive, Bethesda, MD 20892, USA; Reta Lila Weston Research Laboratories, Department of Molecular Neuroscience, UCL Institute of Neurology, London WC1N 3BG, UK), Michael A Nalls (Laboratory of Neurogenetics, National Institute on Aging, National Institutes of Health Building 35, Room 1A215, 35 Convent Drive, Bethesda, MD 20892, USA), Jonathan D Rohrer (Reta Lila Weston Research Laboratories, Department of Molecular Neuroscience, UCL Institute of Neurology, Queen Square, London WC1N 3BG, UK; Dementia Research Centre, Department of Neurodegenerative Disease, UCL Institute of Neurology), Adaikalavan Ramasamy (Reta Lila Weston Research Laboratories, Department of Molecular Neuroscience, UCL Institute of Neurology, London WC1N 3BG, UK; Department of Medical and Molecular Genetics, King’s College London Tower Wing, Guy’s Hospital, London SE1 9RT, UK; The Jenner Institute, University of Oxford, Roosevelt Drive, Oxford OX3 7BQ, UK), John BJ Kwok (Neuroscience Research Australia, Sydney, NSW 2031, Australia; School of Medical Sciences, University of New South Wales, Sydney, NSW 2052, Australia), Carol Dobson-Stone (Neuroscience Research Australia, Sydney, NSW 2031, Australia; School of Medical Sciences, University of New South Wales, Sydney, NSW 2052, Australia), William S Brooks Neuroscience Research Australia, Sydney, NSW 2031, Australia; Prince of Wales Clinical School, University of New South Wales, Sydney, NSW 2052, Australia), Peter R Schofield (Neuroscience Research Australia, Sydney, NSW 2031, Australia; School of Medical Sciences, University of New South Wales, Sydney, NSW 2052, Australia), Glenda M Halliday (Neuroscience Research Australia, Sydney, NSW 2031, Australia; School of Medical Sciences, University of New South Wales, Sydney, NSW 2052, Australia), John R Hodges (Neuroscience Research Australia, Sydney, NSW 2031, Australia; School of Medical Sciences, University of New South Wales, Sydney, NSW 2052, Australia), Olivier Piguet (Neuroscience Research Australia, Sydney, NSW 2031, Australia; School of Medical Sciences, University of New South Wales, Sydney, NSW 2052, Australia), Lauren Bartley (Neuroscience Research Australia, Sydney, NSW 2031, Australia), Elizabeth Thompson (South Australian Clinical Genetics Service, SA Pathology (at Women's and Children's Hospital), North Adelaide, SA 5006, Australia; Department of Paediatrics, University of Adelaide, Adelaide, SA 5000, Australia), Eric Haan (South Australian Clinical Genetics Service, SA Pathology (at Women's and Children's Hospital), North Adelaide, SA 5006, Australia; Department of Paediatrics, University of Adelaide, Adelaide, SA 5000, Australia), Isabel Hernández (Research Center and Memory Clinic of Fundació ACE, Institut Català de Neurociències Aplicades, Barcelona, Spain), Agustín Ruiz (Research Center and Memory Clinic of Fundació ACE, Institut Català de Neurociències Aplicades, Barcelona, Spain), Mercè Boada (Research Center and Memory Clinic of Fundació ACE, Institut Català de Neurociències Aplicades, Barcelona, Spain), Barbara Borroni (Neurology Clinic, University of Brescia, Brescia, Italy), Alessandro Padovani (Neurology Clinic, University of Brescia, Brescia, Italy), Carlos Cruchaga (Department of Psychiatry, Washington University, St. Louis, MO, USA; Hope Center, Washington University School of Medicine, St. Louis, MO, USA), Nigel J Cairns (Hope Center, Washington University School of Medicine, St. Louis, MO, USA; Department of Pathology and Immunology, Washington University, St. Louis, MO, USA), Luisa Benussi (Molecular Markers Laboratory, IRCCS Istituto Centro San Giovanni di Dio Fatebenefratelli,Brescia, Italy), Giuliano Binetti (MAC Memory Clinic, IRCCS Istituto Centro San Giovanni di Dio Fatebenefratelli, Brescia, Italy), Roberta Ghidoni (Molecular Markers Laboratory, IRCCS Istituto Centro San Giovanni di Dio Fatebenefratelli, Brescia, Italy), Gianluigi Forloni (Biology of Neurodegenerative Disorders, IRCCS Istituto di Ricerche Farmacologiche, "Mario Negri", Milano, Italy), Diego Albani (Biology of Neurodegenerative Disorders, IRCCS Istituto di Ricerche Farmacologiche "Mario Negri", Milano, Italy), Daniela Galimberti (University of Milan, Milan, Italy; Fondazione Cà Granda, IRCCS Ospedale Maggiore Policlinico, via F. Sforza 35, 20122, Milan, Italy), Chiara Fenoglio (University of Milan, Milan, Italy; Fondazione Cà Granda, IRCCS Ospedale Maggiore Policlinico, via F. Sforza 35, 20122, Milan, Italy), Maria Serpente (University of Milan, Milan, Italy; Fondazione Cà Granda, IRCCS Ospedale Maggiore Policlinico, via F. Sforza 35, 20122, Milan, Italy), Elio Scarpini (University of Milan, Milan, Italy; Fondazione Cà Granda, IRCCS Ospedale Maggiore Policlinico, via F. Sforza 35, 20122, Milan, Italy), Jordi Clarimón (Memory Unit, Neurology Department and Sant Pau Biomedical Research Institute, Hospital de la Santa Creu i Sant Pau, Universitat Autònoma de Barcelona, Barcelona, Spain; Center for Networker Biomedical Research in Neurodegenerative Diseases (CIBERNED), Madrid, Spain), Alberto Lleó (Memory Unit, Neurology Department and Sant Pau Biomedical Research Institute, Hospital de la Santa Creu i Sant Pau, Universitat Autònoma de Barcelona, Barcelona, Spain; Center for Networker Biomedical Research in Neurodegenerative Diseases (CIBERNED), Madrid, Spain), Rafael Blesa (Memory Unit, Neurology Department and Sant Pau Biomedical Research Institute, Hospital de la Santa Creu i Sant Pau, Universitat Autònoma de Barcelona, Barcelona, Spain; Center for Networker Biomedical Research in Neurodegenerative Diseases (CIBERNED), Madrid, Spain); Maria Landqvist Waldö (Unit of Geriatric Psychiatry, Department of Clinical Sciences, Lund University, Lund, Sweden), Karin Nilsson (Unit of Geriatric Psychiatry, Department of Clinical Sciences, Lund University, Lund, Sweden), Christer Nilsson (Clinical Memory Research Unit, Department of Clinical Sciences, Lund University, Lund, Sweden), Ian RA Mackenzie (Department of Pathology and Laboratory Medicine, University of British Columbia, Vancouver, Canada), Ging-Yuek R Hsiung (Division of Neurology, University of British Columbia, Vancouver, Canada), David MA Mann (Institute of Brain, Behaviour and Mental Health, University of Manchester, Salford Royal Hospital, Stott Lane, Salford, M6 8HD, UK), Jordan Grafman (Rehabilitation Institute of Chicago, Departments of Physical Medicine and Rehabilitation, Psychiatry, and Cognitive Neurology & Alzheimer's Disease Center; Feinberg School of Medicine, Northwestern University, Chicago, USA; Department of Psychology, Weinberg College of Arts and Sciences, Northwestern University, , Chicago, USA), Christopher M Morris (Newcastle Brain Tissue Resource, Institute for Ageing, Newcastle University, NE4 5PL, Newcastle upon Tyne, UK; Newcastle University, Institute of euroscience and Institute for Ageing, Campus for Ageing and Vitality, NE4 5PL, Newcastle upon Tyne, UK; Institute of Neuroscience, Newcastle University Medical School, Framlington Place NE2 4HH, Newcastle upon Tyne, UK), Johannes Attems (Newcastle University, Institute of Neuroscience and Institute for Ageing, Campus for Ageing and Vitality, NE4 5PL, Newcastle upon Tyne, UK; Timothy D Griffiths (Institute of Neuroscience, Newcastle University Medical School, Framlington Place, NE2 4HH, Newcastle upon Tyne, UK), Ian G McKeith (Newcastle University, Institute of Neuroscience and Institute for Ageing, Campus for Ageing and Vitality, Newcastle University, NE4 5PL, Newcastle upon Tyne, UK), Alan J Thomas (Newcastle University, Institute of Neuroscience and Institute for Ageing, Campus for Ageing and Vitality, NE4 5PL, Newcastle upon Tyne, UK), Pietro Pietrini (IMT School for Advanced Studies, Lucca, Lucca, Italy), Edward D Huey (Taub Institute, Departments of Psychiatry and Neurology, Columbia University, 630 West 168th Street New York, NY 10032), Eric M Wassermann (Behavioral Neurology Unit, National Insititute of Neurological Disorders and Stroke, National Insititutes of Health, 10 CENTER DR MSC 1440, Bethesda, MD 20892-1440), Atik Baborie (Department of Laboratory Medicine & Pathology, Walter Mackenzie Health Sciences Centre, 8440 - 112 St, University of Alberta Edmonton, Alberta T6G 2B7, Canada), Evelyn Jaros (Newcastle University, Institute for Ageing and Health, Campus for Ageing and Vitality, NE4 5PL, Newcastle upon Tyne, UK), Michael C Tierney (Behavioral Neurology Unit, National Insititute of Neurological Disorders and Stroke, National Insititutes of Health, 10 CENTER DR MSC 1440, Bethesda, MD 20892-1440), Pau Pastor (Center for Networker Biomedical Research in Neurodegenerative Diseases (CIBERNED), Madrid, Spain; Neurogenetics Laboratory, Division of Neurosciences, Center for Applied Medical Research, Universidad de Navarra, Pamplona, Spain; Department of Neurology, Clínica Universidad de Navarra, University of Navarra School of Medicine, Pamplona, Spain), Cristina Razquin (Neurogenetics Laboratory, Division of Neurosciences, Center for Applied Medical Research, Universidad de Navarra, Pamplona, Spain), Sara Ortega-Cubero (Center for Networker Biomedical Research in Neurodegenerative Diseases (CIBERNED), Madrid, Spain; Neurogenetics Laboratory, Division of Neurosciences, Center for Applied Medical Research, Universidad de Navarra, Pamplona, Spain), Elena Alonso (Neurogenetics Laboratory, Division of Neurosciences, Center for Applied Medical Research, Universidad de Navarra, Pamplona, Spain), Robert Perneczky (Neuroepidemiology and Ageing Research Unit, School of Public Health, Faculty of Medicine, The Imperial College of Science, Technology and Medicine, London W6 8RP, UK; West London Cognitive Disorders Treatment and Research Unit, West London Mental Health Trust, London TW8 8 DS, UK; Department of Psychiatry and Psychotherapy, Technische Universität München, Munich, 81675 Germany), Janine Diehl-Schmid (Department of Psychiatry and Psychotherapy, Technische Universität München, Munich, 81675 Germany), Panagiotis Alexopoulos (Department of Psychiatry and Psychotherapy, Technische Universität München, Munich, 81675 Germany), Alexander Kurz (Department of Psychiatry and Psychotherapy, Technische Universität München, Munich, 81675 Germany), Innocenzo Rainero (Neurology I, Department of Neuroscience, University of Torino, Italy, A.O. Città della Salute e della Scienza di Torino, Torino, Italy), Elisa Rubino (Neurology I, Department of Neuroscience, University of Torino, Italy, A.O. Città della Salute e della Scienza di Torino, Torino, Italy), Lorenzo Pinessi (Neurology I, Department of Neuroscience, University of Torino, Italy, A.O. Città della Salute e della Scienza di Torino, Torino, Italy), Ekaterina Rogaeva (Tanz Centre for Research in Neurodegenerative Diseases, University of Toronto 60 Leonard Street, Toronto, Ontario, Canada, M5T 2S8), Peter St George-Hyslop (Tanz Centre for Research in Neurodegenerative Diseases, University of Toronto, 60 Leonard Street, Toronto, Ontario, Canada, M5T 2S8; Cambridge Institute for Medical Research, and the Department of Clinical Neurosciences, University of Cambridge, Hills Road, Cambridge, UK CB2 0XY), Giacomina Rossi (Division of Neurology V and Neuropathology, Fondazione IRCCS Istituto Neurologico Carlo Besta, 20133 Milano, Italy), Fabrizio Tagliavini (Division of Neurology V and Neuropathology, Fondazione IRCCS Istituto Neurologico Carlo Besta, 20133 Milano, Italy), Giorgio Giaccone (Division of Neurology V and Neuropathology, Fondazione IRCCS Istituto Neurologico Carlo Besta, 20133 Milano, Italy), James B Rowe Cambridge University Department of Clinical Neurosciences, Cambridge, CB2 0SZ, UK; MRC Cognition and Brain Sciences Unit, Cambridge, CB2 7EF, UK; Behavioural and Clinical Neuroscience Institute, Cambridge, CB2 3EB, UK), Johannes CM Schlachetzki (University of California San Diego, Department of Cellular & Molecular Medicine, 9500 Gilman Drive, La Jolla, CA, 92093), James Uphill (MRC Prion Unit, Department of Neurodegenerative Disease, UCL Institute of Neurology, Queen Square House, Queen Square, London, WC1N 3BG), John Collinge (MRC Prion Unit, Department of Neurodegenerative Disease, UCL Institute of Neurology, Queen Square House, Queen Square, London, WC1N 3BG), Simon Mead (MRC Prion Unit, Department of Neurodegenerative Disease, UCL Institute of Neurology, Queen Square House, Queen Square, London, WC1N 3BG), Adrian Danek (Neurologische Klinik und Poliklinik, Ludwig-Maximilians-Universität, Munich, Germany, German Center for Neurodegenerative Diseases (DZNE)), Vivianna M Van Deerlin (University of Pennsylvania Perelman School of Medicine, Department of Pathology and Laboratory Medicine, Philadelphia, PA, USA), Murray Grossman (University of Pennsylvania Perelman School of Medicine, Department of Neurology and Penn Frontotemporal Degeneration Center, Philadelphia, PA, USA), John Q Trojanowski (University of Pennsylvania Perelman School of Medicine, Department of Pathology and Laboratory Medicine, Philadelphia, PA, USA), Julie van der Zee (Neurodegenerative Brain Diseases group, Department of Molecular Genetics, VIB, Antwerp, Belgium; Laboratory of Neurogenetics, Institute Born-Bunge, University of Antwerp, Antwerp, Belgium), Marc Cruts (Neurodegenerative Brain Diseases group, Department of Molecular Genetics, VIB, Antwerp, Belgium; Laboratory of Neurogenetics, Institute Born-Bunge, University of Antwerp, Antwerp, Belgium), Christine Van Broeckhoven (Neurodegenerative Brain Diseases group, Department of Molecular Genetics, VIB, Antwerp, Belgium; Laboratory of Neurogenetics, Institute Born-Bunge, University of Antwerp, Antwerp, Belgium), Stefano F Cappa (Neurorehabilitation Unit, Dept. Of Clinical Neuroscience, Vita-Salute University and San Raffaele Scientific Institute, Milan, Italy), Isabelle Leber (Inserm, UMR_S975, CRICM; UPMC Univ Paris 06, UMR_S975; CNRS UMR 7225, F-75013, Paris, France; AP-HP, Hôpital de la Salpêtrière, Département de neurologie-centre de références des démences rares, F-75013, Paris, France), Didier Hannequin (Service de Neurologie, Inserm U1079, CNR-MAJ, Rouen University Hospital, Rouen, France), Véronique Golfier (Service de neurologie, CH Saint Brieuc, France), Martine Vercelletto (Service de neurologie, CHU Nantes, France), Alexis Brice (Inserm, UMR_S975, CRICM; UPMC Univ Paris 06, UMR_S975; CNRS UMR 7225, F-75013, Paris, France; AP-HP, Hôpital de la Salpêtrière, Département de neurologie-centre de références des démences rares, F-75013, Paris, France), Benedetta Nacmias (Department of Neurosciences, Psychology, Drug Research and Child Health (NEUROFARBA) University of Florence, Florence, Italy), Sandro Sorbi (Department of Neurosciences, Psychology, Drug Research and Child Health (NEUROFARBA), University of Florence and IRCCS "Don Carlo Gnocchi" Firenze, Florence, Italy), Silvia Bagnoli (Department of Neurosciences, Psychology, Drug Research and Child Health (NEUROFARBA), University of Florence, Florence, Italy), Irene Piaceri (Department of Neurosciences, Psychology, Drug Research and Child Health (NEUROFARBA), University of Florence, Florence, Italy), Jørgen E Nielsen (Danish Dementia Research Centre, Neurogenetics Clinic, Department of Neurology, Rigshospitalet, Copenhagen University Hospital, Copenhagen, Denmark; Department of Cellular and Molecular Medicine, Section of Neurogenetics, The Panum Institute, University of Copenhagen, Copenhagen, Denmark), Lena E Hjermind (Danish Dementia Research Centre, Neurogenetics Clinic, Department of Neurology, Rigshospitalet, Copenhagen University Hospital, Copenhagen, Denmark; Department of Cellular and Molecular Medicine, Section of Neurogenetics, The Panum Institute, University of Copenhagen, Copenhagen, Denmark), Matthias Riemenschneider (Saarland University Hospital, Department for Psychiatry & Psychotherapy, Kirrberger Str.1, Bld.90, 66421 Homburg/Saar, Germany; Saarland University, Laboratory for Neurogenetics, Kirrberger Str.1, Bld.90, 66421 Homburg/Saar, Germany), Manuel Mayhaus (Saarland University, Laboratory for Neurogenetics, Kirrberger Str.1, Bld.90, 66421 Homburg/Saar, Germany), Bernd Ibach (University Regensburg, Department of Psychiatry, Psychotherapy and Psychosomatics, Universitätsstr. 84, 93053 Regensburg, Germany), Gilles Gasparoni (Saarland University, Laboratory for Neurogenetics, Kirrberger Str.1, Bld.90, 66421 Homburg/Saar, Germany), Sabrina Pichler (Saarland University, Laboratory for Neurogenetics, Kirrberger Str.1, Bld.90, 66421 Homburg/Saar, Germany), Wei Gu (Saarland University, Laboratory for Neurogenetics, Kirrberger Str.1, Bld.90, 66421 Homburg/Saar, Germany; Luxembourg Centre For Systems Biomedicine (LCSB), University of Luxembourg 7, avenue des Hauts-Fourneaux, 4362 Esch-sur-Alzette, Luxembourg), Martin N Rossor (Dementia Research Centre, Department of Neurodegenerative Disease, UCL Institute of Neurology, Queen Square, London, WC1N 3BG), Nick C Fox (Dementia Research Centre, Department of Neurodegenerative Disease, UCL Institute of Neurology, Queen Square, London, WC1N 3BG), Jason D Warren (Dementia Research Centre, Department of Neurodegenerative Disease, UCL Institute of Neurology, Queen Square, London, WC1N 3BG), Maria Grazia Spillantini (University of Cambridge, Department of Clinical Neurosciences, John Van Geest Brain Repair Centre, Forvie Site, Robinson way, Cambridge CB2 0PY), Huw R Morris (UCL, Department of Molecular Neuroscience, Russell Square House, 9-12 Russell Square House, London, WC1B 5EH), Patrizia Rizzu (German Center for Neurodegenerative Diseases-Tübingen, Otfried Muellerstrasse 23, Tuebingen 72076, Germany), Peter Heutink (German Center for Neurodegenerative Diseases-Tübingen, Otfried Muellerstrasse 23, Tuebingen 72076, Germany), Julie S Snowden (Institute of Brain, Behaviour and Mental Health, Faculty of Medical and Human Sciences, University of Manchester, Manchester, UK), Sara Rollinson (Institute of Brain, Behaviour and Mental Health, Faculty of Medical and Human Sciences, University of Manchester, Manchester, UK), Anna Richardson (Salford Royal Foundation Trust, Faculty of Medical and Human Sciences, University of Manchester, Manchester, UK), Alexander Gerhard (Institute of Brain, Behaviour and Mental Health, The University of Manchester, 27 Palatine Road, Withington, Manchester, M20 3LJ, UK), Amalia C Bruni (Regional Neurogenetic Centre, ASPCZ, Lamezia Terme, Italy), Raffaele Maletta (Regional Neurogenetic Centre, ASPCZ, Lamezia Terme, Italy), Francesca Frangipane (Regional Neurogenetic Centre, ASPCZ, Lamezia Terme, Italy), Chiara Cupidi (Regional Neurogenetic Centre, ASPCZ, Lamezia Terme, Italy), Livia Bernardi (Regional Neurogenetic Centre, ASPCZ, Lamezia Terme, Italy), Maria Anfossi (Regional Neurogenetic Centre, ASPCZ, Lamezia Terme, Italy), Maura Gallo (Regional Neurogenetic Centre, ASPCZ, Lamezia Terme, Italy), Maria Elena Conidi Regional Neurogenetic Centre, ASPCZ, Lamezia Terme, Italy), Nicoletta Smirne (Regional Neurogenetic Centre, ASPCZ, Lamezia Terme, Italy), Rosa Rademakers (Department of Neuroscience, Mayo Clinic Jacksonville, 4500 San Pablo Road, Jacksonville, FL 32224), Matt Baker (Department of Neuroscience, Mayo Clinic Jacksonville, 4500 San Pablo Road, Jacksonville, FL 32224), Dennis W Dickson (Department of Neuroscience, Mayo Clinic Jacksonville, 4500 San Pablo Road, Jacksonville, FL 32224), Neill R Graff-Radford (Department of Neurology, Mayo Clinic Jacksonville, 4500 San Pablo Road, Jacksonville, FL 32224), Ronald C Petersen (Department of Neurology, Mayo Clinic Rochester, 2001st street SW Rochester MN 5905), David Knopman (Department of Neurology, Mayo Clinic Rochester, 2001st street SW Rochester MN 5905), Keith A Josephs (Department of Neurology, Mayo Clinic Rochester, 2001st street SW Rochester MN 5905), Bradley F Boeve (Department of Neurology, Mayo Clinic Rochester, 2001st street SW Rochester MN 5905), Joseph E Parisi (Department of Pathology, Mayo Clinic Rochester, 2001st street SW Rochester MN 5905), William W Seeley (Department of Neurology, Box 1207, University of California, San Francisco, CA 94143, USA), Bruce L Miller (Memory and Aging Center, Department of Neurology, University of California, San Francisco, CA 94158, USA), Anna M Karydas (Memory and Aging Center, Department of Neurology, University of California, San Francisco, CA 94158, USA), Howard Rosen (Memory and Aging Center, Department of Neurology, University of California, San Francisco, CA 94158, USA), John C van Swieten (Department of Neurology, Erasmus Medical Centre, Rotterdam, The Netherlands; Department of Medical Genetics, VU university Medical Centre, Amsterdam, The Netherlands), Elise GP Dopper (Department of Neurology, Erasmus Medical Centre, Rotterdam, The Netherlands), Harro Seelaar (Department of Neurology, Erasmus Medical Centre, Rotterdam, The Netherlands), Yolande AL Pijnenburg (Alzheimer Centre and department of neurology, VU University medical centre, Amsterdam, The Netherlands), Philip Scheltens (Alzheimer Centre and department of neurology, VU University medical centre, Amsterdam, The Netherlands), Giancarlo Logroscino (Department of Basic Medical Sciences, Neurosciences and Sense Organs of the "Aldo Moro" University of Bari, Bari, Italy), Rosa Capozzo (Department of Basic Medical Sciences, Neurosciences and Sense Organs of the "Aldo Moro" University of Bari, Bari, Italy), Valeria Novelli (Medical Genetics Unit, Fondazione Policlinico Universitario A. Gemelli, Rome, Italy), Annibale A Puca (Cardiovascular Research Unit, IRCCS Multimedica, Milan, Italy; Department of Medicine and Surgery, University of Salerno, Baronissi (SA), Italy), Massimo Franceschi (Neurology Dept, IRCCS Multimedica, Milan, Italy), Alfredo Postiglione (Department of Clinical Medicine and Surgery, University of Naples Federico II, Naples, Italy), Graziella Milan (Geriatric Center Frullone- ASL Napoli 1 Centro, Naples, Italy), Paolo Sorrentino (Geriatric Center Frullone- ASL Napoli 1 Centro, Naples, Italy), Mark Kristiansen (UCL Genomics, Institute of Child Health (ICH), UCL, London, UK), Huei-Hsin Chiang (Karolinska Institutet, Dept NVS, Alzheimer Research Center, Novum, SE-141 57, Stockholm, Sweden; Dept of Geriatric Medicine, Genetics Unit, M51, Karolinska University Hospital, SE-14186, Stockholm), Caroline Graff (Karolinska Institutet, Dept NVS, Alzheimer Research Center, Novum, SE-141 57, Stockholm, Sweden; Dept of Geriatric Medicine, Genetics Unit, M51, Karolinska University Hospital, SE-14186, Stockholm), Florence Pasquier (Univ Lille, Inserm 1171, DISTALZ, CHU 59000 Lille, France), Adeline Rollin (Univ Lille, Inserm 1171, DISTALZ, CHU 59000 Lille, France), Vincent Deramecourt (Univ Lille, Inserm 1171, DISTALZ, CHU 59000 Lille, France), Thibaud Lebouvier (Univ Lille, Inserm 1171, DISTALZ, CHU 59000 Lille, France), Dimitrios Kapogiannis (National Institute on Aging (NIA/NIH), 3001 S. Hanover St, NM 531, Baltimore, MD, 21230), Luigi Ferrucci (Clinical Research Branch, National Institute on Aging, Baltimore, MD, USA), Stuart Pickering-Brown (Institute of Brain, Behaviour and Mental Health, Faculty of Medical and Human Sciences, University of Manchester, Manchester, UK), Andrew B Singleton (Laboratory of Neurogenetics, National Institute on Aging, National Institutes of Health, Building 35, Room 1A215, 35 Convent Drive, Bethesda, MD 20892, USA), John Hardy (UCL, Department of Molecular Neuroscience, Russell Square House, 9-12 Russell Square House, London, WC1B 5EH), Parastoo Momeni (Laboratory of Neurogenetics, Department of Internal Medicine, Texas Tech University Health Science Center, 4th street, Lubbock, Texas 79430, USA)

**The International Parkinson’s Disease Genomics Consortium (IPDGC)**

**Acknowledgments**

We would like to thank all of the subjects who donated their time and biological samples to be a part of this study.

This work was supported in part by the Intramural Research Programs of the National Institute of Neurological Disorders and Stroke (NINDS), the National Institute on Aging (NIA), and the National Institute of Environmental Health Sciences both part of the National Institutes of Health, Department of Health and Human Services; project numbers Z01-AG000949-02 and Z01-ES101986. In addition this work was supported by the Department of Defense (award W81XWH-09-2-0128), and The Michael J Fox Foundation for Parkinson’s Research. This work was supported by National Institutes of Health grants R01NS037167, R01CA141668, P50NS071674, American Parkinson Disease Association (APDA); Barnes Jewish Hospital Foundation; Greater St Louis Chapter of the APDA; Hersenstichting Nederland; Neuroscience Campus Amsterdam; and the section of medical genomics, the Prinses Beatrix Fonds. The KORA (Cooperative Research in the Region of Augsburg) research platform was started and financed by the Forschungszentrum für Umwelt und Gesundheit, which is funded by the German Federal Ministry of Education, Science, Research, and Technology and by the State of Bavaria. This study was also funded by the German National Genome Network (NGFNplus number 01GS08134, German Ministry for Education and Research); by the German Federal Ministry of Education and Research (NGFN 01GR0468, PopGen); and 01EW0908 in the frame of ERA-NET NEURON and Helmholtz Alliance Mental Health in an Ageing Society (HA-215), which was funded by the Initiative and Networking Fund of the Helmholtz Association. The French GWAS work was supported by the French National Agency of Research (ANR-08-MNP-012). This study was also funded by France-Parkinson Association, the French program “Investissements d’avenir” funding (ANR-10-IAIHU-06) and a grant from Assistance Publique-Hôpitaux de Paris (PHRC, AOR-08010) for the French clinical data. This study was also sponsored by the Landspitali University Hospital Research Fund (grant to SSv); Icelandic Research Council (grant to SSv); and European Community Framework Programme 7, People Programme, and IAPP on novel genetic and phenotypic markers of Parkinson’s disease and Essential Tremor (MarkMD), contract number PIAP-GA-2008-230596 MarkMD (to HP and JHu). This study utilized the high-performance computational capabilities of the Biowulf Linux cluster at the National Institutes of Health, Bethesda, Md. (http://biowulf.nih.gov), and DNA panels, samples, and clinical data from the National Institute of Neurological Disorders and Stroke Human Genetics Resource Center DNA and Cell Line Repository. People who contributed samples are acknowledged in descriptions of every panel on the repository website. We thank the French Parkinson’s Disease Genetics Study Group and the Drug Interaction with genes (DIGPD) study group: Y Agid, M Anheim, A-M Bonnet, M Borg, A Brice, E Broussolle, J-C Corvol, P Damier, A Destée, A Dürr, F Durif, A Elbaz, D Grabil, S Klebe, P. Krack, E Lohmann, L. Lacomblez, M Martinez, V Mesnage, P Pollak, O Rascol, F Tison, C Tranchant, M Vérin, F Viallet, and M Vidailhet. We also thank the members of the French 3C Consortium: A Alpérovitch, C Berr, C Tzourio, and P Amouyel for allowing us to use part of the 3C cohort, and D Zelenika for support in generating the genome-wide molecular data. We thank P Tienari (Molecular Neurology Programme, Biomedicum, University of Helsinki), T Peuralinna (Department of Neurology, Helsinki University Central Hospital), L Myllykangas (Folkhalsan Institute of Genetics and Department of Pathology, University of Helsinki), and R Sulkava (Department of Public Health and General Practice Division of Geriatrics, University of Eastern Finland) for the Finnish controls (Vantaa85+ GWAS data). We used genome-wide association data generated by the Wellcome Trust Case-Control Consortium 2 (WTCCC2) from UK patients with Parkinson’s disease and UK control individuals from the 1958 Birth Cohort and National Blood Service. Genotyping of UK replication cases on ImmunoChip was part of the WTCCC2 project, which was funded by the Wellcome Trust (083948/Z/07/Z). UK population control data was made available through WTCCC1. This study was supported by the Medical Research Council and Wellcome Trust disease centre (grant WT089698/Z/09/Z to NW, JHa, and ASc). As with previous IPDGC efforts, this study makes use of data generated by the Wellcome Trust Case-Control Consortium. A full list of the investigators who contributed to the generation of the data is available from www.wtccc.org.uk. Funding for the project was provided by the Wellcome Trust under award 076113, 085475 and 090355. This study was also supported by Parkinson’s UK (grants 8047 and J-0804) and the Medical Research Council (G0700943). We thank Jeffrey Barrett for assistance with the design of the ImmunoChip. DNA extraction work that was done in the UK was undertaken at University College London Hospitals, University College London, who received a proportion of funding from the Department of Health’s National Institute for Health Research Biomedical Research Centres funding. This study was supported in part by the Wellcome Trust/Medical Research Council Joint Call in Neurodegeneration award (WT089698) to the Parkinson’s Disease Consortium (UKPDC), whose members are from the UCL Institute of Neurology, University of Sheffield, and the Medical Research Council Protein Phosphorylation Unit at the University of Dundee.

**IPDGC members and affiliations**: Mike A Nalls (Laboratory of Neurogenetics, National Institute on Aging, National Institutes of Health, Bethesda, MD, USA), Vincent Plagnol (UCL Genetics Institute, London, UK), Dena G Hernandez (Laboratory of Neurogenetics, National Institute on Aging; and Department of Molecular Neuroscience, UCL Institute of Neurology, London, UK), Manu Sharma (Department for Neurodegenerative Diseases, Hertie Institute for Clinical Brain Research, University of Tübingen, and DZNE, German Center for Neurodegenerative Diseases, Tübingen, Germany), Una-Marie Sheerin (Department of Molecular Neuroscience, UCL Institute of Neurology), Mohamad Saad (INSERM U563, CPTP, Toulouse, France; and Paul Sabatier University, Toulouse, France), Javier Simón-Sánchez (Department of Clinical Genetics, Section of Medical Genomics, VU University Medical Centre, Amsterdam, Netherlands), Claudia Schulte (Department for Neurodegenerative Diseases, Hertie Institute for Clinical Brain Research), Suzanne Lesage (INSERM, UMR_S975 [ formerly UMR_S679], Paris, France; Université Pierre et Marie Curie-Paris, Centre de Recherche de l’Institut du Cerveau et de la Moelle épinière, Paris, France; and CNRS, Paris, France), Sigurlaug Sveinbjörnsdóttir (Department of Neurology, Landspítali University Hospital, Reykjavík, Iceland; Department of Neurology, MEHT Broomfield Hospital, Chelmsford, Essex, UK; and Queen Mary College, University of London, London, UK), Sampath Arepalli (Laboratory of Neurogenetics, National Institute on Aging), Roger Barker (Department of Neurology, Addenbrooke’s Hospital, University of Cambridge, Cambridge, UK), Yoav Ben-Shlomo (School of Social and Community Medicine, University of Bristol), Henk W Berendse (Department of Neurology and Alzheimer Center, VU University Medical Center), Daniela Berg (Department for Neurodegenerative Diseases, Hertie Institute for Clinical Brain Research and DZNE, German Center for Neurodegenerative diseases), Kailash Bhatia (Department of Motor Neuroscience, UCL Institute of Neurology), Rob M A de Bie (Department of Neurology, Academic Medical Center, University of Amsterdam, Amsterdam, Netherlands), Alessandro Biffi (Center for Human Genetic Research and Department of Neurology, Massachusetts General Hospital, Boston, MA, USA; and Program in Medical and Population Genetics, Broad Institute, Cambridge, MA, USA), Bas Bloem (Department of Neurology, Radboud University Nijmegen Medical Centre, Nijmegen, Netherlands), Zoltan Bochdanovits (Department of Clinical Genetics, Section of Medical Genomics, VU University Medical Centre), Michael Bonin (Department of Medical Genetics, Institute of Human Genetics, University of Tübingen, Tübingen, Germany), Jose M Bras (Department of Molecular Neuroscience, UCL Institute of Neurology), Kathrin Brockmann (Department for Neurodegenerative Diseases, Hertie Institute for Clinical Brain Research and DZNE, German Center for Neurodegenerative diseases), Janet Brooks (Laboratory of Neurogenetics, National Institute on Aging), David J Burn (Newcastle University Clinical Ageing Research Unit, Campus for Ageing and Vitality, Newcastle upon Tyne, UK), Gavin Charlesworth (Department of Molecular Neuroscience, UCL Institute of Neurology), Honglei Chen (Epidemiology Branch, National Institute of Environmental Health Sciences, National Institutes of Health, NC, USA), Patrick F Chinnery (Neurology M4104, The Medical School, Framlington Place, Newcastle upon Tyne, UK), Sean Chong (Laboratory of Neurogenetics, National Institute on Aging), Carl E Clarke (School of Clinical and Experimental Medicine, University of Birmingham, Birmingham, UK; and Department of Neurology, City Hospital, Sandwell and West Birmingham Hospitals NHS Trust, Birmingham, UK), Mark R Cookson (Laboratory of Neurogenetics, National Institute on Aging), J Mark Cooper (Department of Clinical Neurosciences, UCL Institute of Neurology), Jean Christophe Corvol (INSERM, UMR_S975; Université Pierre et Marie Curie-Paris; CNRS; and INSERM CIC-9503, Hôpital Pitié-Salpêtrière, Paris, France), Carl Counsell (University of Aberdeen, Division of Applied Health Sciences, Population Health Section, Aberdeen, UK), Philippe Damier (CHU Nantes, CIC0004, Service de Neurologie, Nantes, France), Jean-François Dartigues (INSERM U897, Université Victor Segalen, Bordeaux, France), Panos Deloukas (Wellcome Trust Sanger Institute, Wellcome Trust Genome Campus, Cambridge, UK), Günther Deuschl (Klinik für Neurologie, Universitätsklinikum Schleswig-Holstein, Campus Kiel, Christian-Albrechts-Universität Kiel, Kiel, Germany), David T Dexter (Parkinson’s Disease Research Group, Faculty of Medicine, Imperial College London, London, UK), Karin D van Dijk (Department of Neurology and Alzheimer Center, VU University Medical Center), Allissa Dillman (Laboratory of Neurogenetics, National Institute on Aging), Frank Durif (Service de Neurologie, Hôpital Gabriel Montpied, Clermont-Ferrand, France), Alexandra Dürr (INSERM, UMR_S975; Université Pierre et Marie Curie-Paris; CNRS; and AP-HP, Pitié-Salpêtrière Hospital), Sarah Edkins (Wellcome Trust Sanger Institute), Jonathan R Evans (Cambridge Centre for Brain Repair, Cambridge, UK), Thomas Foltynie (UCL Institute of Neurology), Jing Dong (Epidemiology Branch, National Institute of Environmental Health Sciences), Michelle Gardner (Department of Molecular Neuroscience, UCL Institute of Neurology), J Raphael Gibbs (Laboratory of Neurogenetics, National Institute on Aging; and Department of Molecular Neuroscience, UCL Institute of Neurology), Alison Goate (Department of Psychiatry, Department of Neurology, Washington University School of Medicine, MI, USA), Emma Gray (Wellcome Trust Sanger Institute), Rita Guerreiro (Department of Molecular Neuroscience, UCL Institute of Neurology), Clare Harris (University of Aberdeen), Jacobus J van Hilten (Department of Neurology, Leiden University Medical Center, Leiden, Netherlands), Albert Hofman (Department of Epidemiology, Erasmus University Medical Center, Rotterdam, Netherlands), Albert Hollenbeck (AARP, Washington DC, USA), Janice Holton (Queen Square Brain Bank for Neurological Disorders, UCL Institute of Neurology), Michele Hu (Department of Clinical Neurology, John Radcliffe Hospital, Oxford, UK), Xuemei Huang (Departments of Neurology, Radiology, Neurosurgery, Pharmacology, Kinesiology, and Bioengineering, Pennsylvania State University– Milton S Hershey Medical Center, Hershey, PA, USA), Isabel Wurster (Department for Neurodegenerative Diseases, Hertie Institute for Clinical Brain Research and German Center for Neurodegenerative diseases), Walter Mätzler (Department for Neurodegenerative Diseases, Hertie Institute for Clinical Brain Research and German Center for Neurodegenerative diseases), Gavin Hudson (Neurology M4104, The Medical School, Newcastle upon Tyne, UK), Sarah E Hunt (Wellcome Trust Sanger Institute), Johanna Huttenlocher (deCODE genetics), Thomas Illig (Institute of Epidemiology, Helmholtz Zentrum München, German Research Centre for Environmental Health, Neuherberg, Germany), Pálmi V Jónsson (Department of Geriatrics, Landspítali University Hospital, Reykjavík, Iceland), Jean-Charles Lambert (INSERM U744, Lille, France; and Institut Pasteur de Lille, Université de Lille Nord, Lille, France), Cordelia Langford (Cambridge Centre for Brain Repair), Andrew Lees (Queen Square Brain Bank for Neurological Disorders), Peter Lichtner (Institute of Human Genetics, Helmholtz Zentrum München, German Research Centre for Environmental Health, Neuherberg, Germany), Patricia Limousin (Institute of Neurology, Sobell Department, Unit of Functional Neurosurgery, London, UK), Grisel Lopez (Section on Molecular Neurogenetics, Medical Genetics Branch, NHGRI, National Institutes of Health), Delia Lorenz (Klinik für Neurologie, Universitätsklinikum Schleswig-Holstein), Alisdair McNeill (Department of Clinical Neurosciences, UCL Institute of Neurology), Catriona Moorby (School of Clinical and Experimental Medicine, University of Birmingham), Matthew Moore (Laboratory of Neurogenetics, National Institute on Aging), Huw R Morris (MRC Centre for Neuropsychiatric Genetics and Genomics, Cardiff University School of Medicine, Cardiff, UK), Karen E Morrison (School of Clinical and Experimental Medicine, University of Birmingham; and Neurosciences Department, Queen Elizabeth Hospital, University Hospitals Birmingham NHS Foundation Trust, Birmingham, UK), Ese Mudanohwo (Neurogenetics Unit, UCL Institute of Neurology and National Hospital for Neurology and Neurosurgery), Sean S O’Sullivan (Queen Square Brain Bank for Neurological Disorders), Justin Pearson (MRC Centre for Neuropsychiatric Genetics and Genomics), Joel S Perlmutter (Department of Neurology, Radiology, and Neurobiology at Washington University, St Louis), Hjörvar Pétursson (deCODE genetics; and Department of Medical Genetics, Institute of Human Genetics, University of Tübingen), Pierre Pollak (Service de Neurologie, CHU de Grenoble, Grenoble, France), Bart Post (Department of Neurology, Radboud University Nijmegen Medical Centre), Simon Potter (Wellcome Trust Sanger Institute), Bernard Ravina (Translational Neurology, Biogen Idec, MA, USA), Tamas Revesz (Queen Square Brain Bank for Neurological Disorders), Olaf Riess (Department of Medical Genetics, Institute of Human Genetics, University of Tübingen), Fernando Rivadeneira (Departments of Epidemiology and Internal Medicine, Erasmus University Medical Center), Patrizia Rizzu (Department of Clinical Genetics, Section of Medical Genomics, VU University Medical Centre), Mina Ryten (Department of Molecular Neuroscience, UCL Institute of Neurology), Stephen Sawcer (University of Cambridge, Department of Clinical Neurosciences, Addenbrooke’s hospital, Cambridge, UK), Anthony Schapira (Department of Clinical Neurosciences, UCL Institute of Neurology), Hans Scheffer (Department of Human Genetics, Radboud University Nijmegen Medical Centre, Nijmegen, Netherlands), Karen Shaw (Queen Square Brain Bank for Neurological Disorders), Ira Shoulson (Department of Neurology, University of Rochester, Rochester, NY, USA), Ellen Sidransky (Section on Molecular Neurogenetics, Medical Genetics Branch, NHGRI), Colin Smith (Department of Pathology, University of Edinburgh, Edinburgh, UK), Chris C A Spencer (Wellcome Trust Centre for Human Genetics, Oxford, UK), Hreinn Stefánsson (deCODE genetics), Francesco Bettella (deCODE genetics), Joanna D Stockton (School of Clinical and Experimental Medicine), Amy Strange (Wellcome Trust Centre for Human Genetics), Kevin Talbot (University of Oxford, Department of Clinical Neurology, John Radcliffe Hospital, Oxford, UK), Carlie M Tanner (Clinical Research Department, The Parkinson’s Institute and Clinical Center, Sunnyvale, CA, USA), Avazeh Tashakkori-Ghanbaria (Wellcome Trust Sanger Institute), François Tison (Service de Neurologie, Hôpital Haut-Lévêque, Pessac, France), Daniah Trabzuni (Department of Molecular Neuroscience, UCL Institute of Neurology), Bryan J Traynor (Laboratory of Neurogenetics, National Institute on Aging), André G Uitterlinden (Departments of Epidemiology and Internal Medicine, Erasmus University Medical Center), Daan Velseboer (Department of Neurology, Academic Medical Center), Marie Vidailhet (INSERM, UMR_S975, Université Pierre et Marie Curie-Paris, CNRS, UMR 7225), Robert Walker (Department of Pathology, University of Edinburgh), Bart van de Warrenburg (Department of Neurology, Radboud University Nijmegen Medical Centre), Mirdhu Wickremaratchi (Department of Neurology, Cardiff University, Cardiff, UK), Nigel Williams (MRC Centre for Neuropsychiatric Genetics and Genomics), Caroline H Williams-Gray (Department of Neurology, Addenbrooke’s Hospital), Sophie Winder-Rhodes (Department of Psychiatry and Medical Research Council and Wellcome Trust Behavioural and Clinical Neurosciences Institute, University of Cambridge), Kári Stefánsson (deCODE genetics), Maria Martinez (INSERM UMR 1043; and Paul Sabatier University), Nicholas W Wood (UCL Genetics Institute; and Department of Molecular Neuroscience, UCL Institute of Neurology), John Hardy (Department of Molecular Neuroscience, UCL Institute of Neurology), Peter Heutink (Department of Clinical Genetics, Section of Medical Genomics, VU University Medical Centre), Alexis Brice (INSERM, UMR_S975, Université Pierre et Marie Curie-Paris, CNRS, UMR 7225, AP-HP, Pitié-Salpêtrière Hospital), Thomas Gasser (Department for Neurodegenerative Diseases, Hertie Institute for Clinical Brain Research, and DZNE, German Center for Neurodegenerative Diseases), Andrew B Singleton (Laboratory of Neurogenetics, National Institute on Aging).

**Mayo RNAseq**: Study data were provided by the following sources: The Mayo Clinic Alzheimers Disease Genetic Studies, led by Dr. Nilufer Taner and Dr. Steven G. Younkin, Mayo Clinic, Jacksonville, FL using samples from the Mayo Clinic Study of Aging, the Mayo Clinic Alzheimers Disease Research Center, and the Mayo Clinic Brain Bank. Data collection was supported through funding by NIA grants P50 AG016574, R01 AG032990, U01 AG046139, R01 AG018023, U01 AG006576, U01 AG006786, R01 AG025711, R01 AG017216, R01 AG003949, NINDS grant R01 NS080820, CurePSP Foundation, and support from Mayo Foundation. Study data includes samples collected through the Sun Health Research Institute Brain and Body Donation Program of Sun City, Arizona. The Brain and Body Donation Program is supported by the National Institute of Neurological Disorders and Stroke (U24 NS072026 National Brain and Tissue Resource for Parkinsons Disease and Related Disorders), the National Institute on Aging (P30 AG19610 Arizona Alzheimers Disease Core Center), the Arizona Department of Health Services (contract 211002, Arizona Alzheimers Research Center), the Arizona Biomedical Research Commission (contracts 4001, 0011, 05-901 and 1001 to the Arizona Parkinson's Disease Consortium) and the Michael J. Fox Foundation for Parkinsons Research.

**MSBB**: These data were generated from postmortem brain tissue collected through the Mount Sinai VA Medical Center Brain Bank and were provided by Dr. Eric Schadt from Mount Sinai School of Medicine.
